# Obesity Has a Systemic Effect on Immune Cells in Naïve and Cancer-Bearing Mice

**DOI:** 10.3390/ijms22168803

**Published:** 2021-08-16

**Authors:** Silke Neumann, Katrin Campbell, Matthew J. Woodall, Meghan Evans, Andrew N. Clarkson, Sarah L. Young

**Affiliations:** 1 Department of Pathology, Dunedin School of Medicine, University of Otago, Dunedin 9016, New Zealand; silke.neumann@otago.ac.nz (S.N.); katrin.campbell@yahoo.com (K.C.); wooma060@student.otago.ac.nz (M.J.W.); meghan.evans257@gmail.com (M.E.); 2 Brain Health Research Centre and Brain Research New Zealand, Department of Anatomy, University of Otago, Dunedin 9016, New Zealand; andrew.clarkson@otago.ac.nz; 3 Faculty of Medicine and Health, School of Medical Sciences, The University of Sydney, Sydney 2006, Australia

**Keywords:** obesity, breast cancer, colorectal cancer, myeloid cells, tumour-infiltrating lymphocytes, inflammation, tumour microenvironment

## Abstract

Obesity is a major risk factor for developing cancer, with obesity-induced immune changes and inflammation in breast (BC) and colorectal cancer (CRC) providing a potential link between the two. This study investigates systemic effects of obesity on adaptive and innate immune cells in healthy and tumour-bearing mice. Immune cells from lean and obese mice were phenotyped prior to implantation of either BC (C57mg and EO771.LMB) or CRC (MC38) cells as tumour models. Tumour growth rate, tumour-infiltrating lymphocytes (TIL) and peripheral blood immune cell populations were compared between obese and lean mice. In vitro studies showed that naïve obese mice had higher levels of myeloid cells in the bone marrow and bone marrow-derived dendritic cells expressed lower levels of activation markers compared to cells from their lean counterparts. In the tumour setting, BC tumours grew faster in obese mice than in lean mice and lower numbers of TILs as well as higher frequency of exhausted T cells were observed. Data from peripheral blood showed lower levels of myeloid cells in tumour-bearing obese mice. This study highlights that systemic changes to the immune system are relevant for tumour burden and provides a potential mechanism behind the effects of obesity on cancer development and progression in patients.

## 1. Introduction

Obesity is one of the most serious health problems worldwide, with obesity rates having tripled over recent decades. Generally defined by a Body Mass Index (BMI) of ≥30 by the World Health Organisation, obesity is caused by an energy surplus that favours weight gain, resulting in metabolic disturbances causing stress to cells and tissues [1]. The prevalence of obesity-associated conditions such as cardiovascular disease, type-2 diabetes mellitus, osteoarthritis and psychological problems has risen in both adults and children [2,3]. Cancer is one of the leading causes of death worldwide and cancer rates are expected to increase by over 40% by 2030. Carcinogenesis, the formation of cancer, is a process with many influencers including genetic, environmental and lifestyle factors which all contribute to the development and malignant progression of the disease. Overweight and obesity are a major risk factor for developing cancer, and an increase in mortality from cancer has been related to obesity [4,5].

Epidemiological studies have shown that breast cancer (BC) and colorectal cancer (CRC), among others, have been associated with obesity [6]. Overall, obesity is the second highest risk factor for cancer, with the number of cases of cancer estimated to be caused by obesity being 20% [7]. The reasons for why obesity has been associated with a higher cancer risk in general and specifically in BC and CRC, is not fully elucidated, but it has been shown to relate to obesity-induced immune changes and inflammation [5]. Inflammation and tumour development have long been linked, with chronic inflammation increasing the risk of individuals to develop various types of cancers [8]. The metabolic syndrome is classified as a cluster of disorders that increase the risk of cardiovascular disease, and often occur together due to a shared chronic inflammatory state. The main criteria include obesity, hyperglycaemia, hypertension and dyslipidaemia [9]. The large lipid droplet inside hypertrophic adipocytes leads to pressure on the plasma membrane to expand, thereby inducing shear mechanical stress on the extracellular environment. This enhanced strain activates stress responses in the endoplasmic reticulum and mitochondria, leading to a pro-inflammatory state within the adipose tissue [1,10,11]. This persistent state of inflammation leads to apoptosis of adipocytes, triggering an infiltration of inflammatory leukocytes. The enhanced numbers of inflammatory leukocytes then leads to an increase in macrophage numbers within adipose tissue, which encircle the dead adipocytes to form crown-like structures (CLS) [12,13,14]. Both inflammatory macrophages and adipocytes upregulate the secretion of pro-inflammatory mediators, such as tumour necrosis factor (TNF)α, interleukin (IL)-1, IL-6, interferon (IFN)-y, monocyte chemoattractant protein-1 and leptin [15,16]. High levels of leptin, which normally acts to suppress food intake, can promote mitosis and are pro-inflammatory, anti-apoptotic and pro-angiogenic by synergistically acting with VEGF [17]. Prostate, colon, and breast cancers have all been associated with an increase in leptin concentration [18,19,20,21].

Leukocytes circulating in the blood demonstrate a basal pro-inflammatory state that is also observed in adipose tissue [22]. The pro-inflammatory state is characterized by increased activation of transcription factor nuclear factor κB (NF-κB), an important regulator of inflammasome activity and cytokine and chemokine production. In the adipose tissue, increases in proportions of immune-suppressive innate immune populations, neutrophils, dendritic cells, natural killer cells, mast cells, B-cells, Th1 CD4^+^ T cells and CD8^+^ T cells have been observed in obese patients [23]. However, multiple studies have failed to find consistent differences in the cell frequency and cytokine profile of non-obese and obese patients that matches the current theories, highlighting the complexity of the inflammatory changes brought about by obesity [24,25,26].

Another studied link between obesity and cancer incidence is immune escape, which is characterised by the immune system being unable to respond to and eliminate tumour cells. It is a complex process caused by tumour cells losing their immunogenicity, losing their antigenicity, the immunosuppressive nature of the tumour microenvironment (TME), or a combination of all three [27]. Tumour-infiltrating lymphocytes (TILs) are T cells that have migrated into the tumour mass, and play an important role in targeting tumour cells or inducing immunosuppression [28]. A hallmark of T cell exhaustion is the enhanced expression of inhibitory checkpoint receptors such as PD-1, Tim-3, CD127 and LAG-3 [29]. In tumours from obese patients, lower frequencies, as well as less active adaptive TILs, have been reported, resulting in a reduced T-cell receptor repertoire of circulating T cells, thereby reducing the number of antigens that can be recognised [10]. This reduced capability of recognising antigens, as well as an increase in exhausted T cells in tumours from obese patients, can result in an enhanced probability of immune escape of cancer in obese patients [30].

However, while these findings point to a link between obesity and in increased risk of developing cancers in general and BC and CRC in specific, the specific mechanisms have not been fully elucidated. Here we investigated systemic effects of obesity on innate and adaptive immune cell populations in healthy and tumour-bearing mice as well as changes in tumour growth characteristics in order to delineate how obesity both increases the susceptibility to developing cancer, as well as influences the behaviour of the tumours as they develop.

## 2. Results

### 2.1. Bone Marrow Cell Characterisation

**Figure 1:**
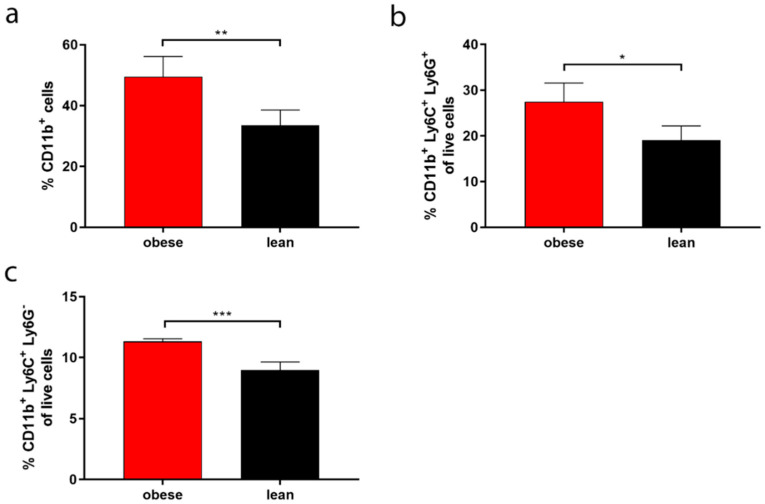
Obese mice had elevated levels of myeloid cells in the bone marrow as compared with lean mice. Bone marrow cells were isolated from obese and lean mice and analysed for the frequency of (**a**) CD11b^+^ cells; (**b**) CD11b^+^ Ly6C^+^ Ly6G^+^ cells (inflammatory monocytes) and (**c**) CD11b^+^ Ly6C^+^ Ly6G^−^ cells (neutrophils) by flow cytometry. Frequencies are shown as a percentage of live cells. Data are the mean + STD from four independent repeats, with 1 female mouse/repeat. Unpaired student’s *t*-test: *** *p* < 0.001, ** *p* < 0.05, * *p* < 0.01.

Initial experiments aimed to characterise phenotypic differences in bone marrow (BM) cells between lean and obese mice. Flow cytometry was used to determine the expression of the cell surface markers CD11b, Ly6C and Ly6G on BM cells commonly used to identify myeloid cells. Cells in the BM of obese mice contained a significantly higher frequency of CD11b^+^ cells as compared to cells in the BM of lean mice, with mean frequencies of 49.6% and 33.5%, respectively (Figure 1a). A significantly higher frequency of cells in the BM of obese mice were characterised as CD11b^+^ Ly6C^+^, Ly6G^−^ (inflammatory monocytes) with a mean frequency of 11.3% as compared with 9.0% for inflammatory monocytes of lean mice (Figure 1c). Comparably, the frequency of CD11b^+^ Ly6C^+^, Ly6G^−^ neutrophils was significantly higher in obese mice as opposed to lean mice, with mean frequencies of 27.5% and 19.1%, respectively (Figure 1b).

### 2.2. Bone Marrow-Derived Dendritic Cell Activation

To characterise differences in activation markers on BMDCs from obese and lean mice, BMDCs were left un-stimulated or stimulated with CpG (0.25 nmol/mL) and analysed by flow cytometry for their expression of MHC II and the co-stimulatory molecules CD40, CD80 and CD86, using the cell surface marker CD11c to identify BMDCs (Figure S2).

**Figure 2:**
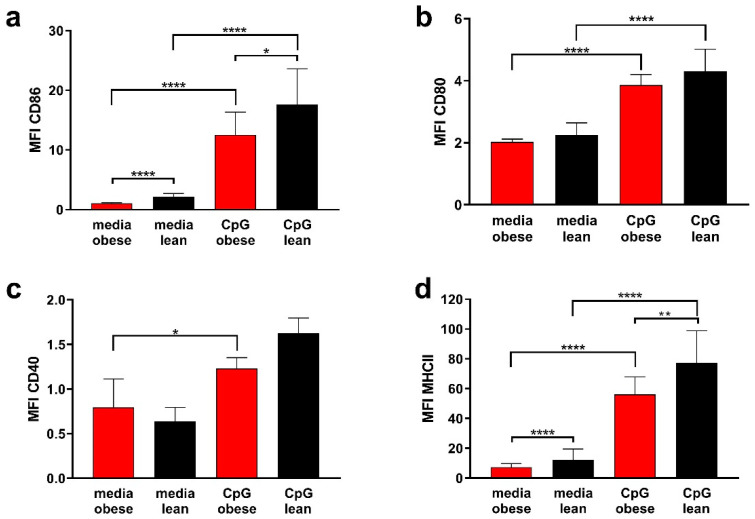
BMDCs generated from the bone marrow of obese and lean mice differ in the expression of activation markers (**a**) CD86 and (**d**) MHCII after stimulation with the vaccine adjuvant CpG. Expression of activation markers (**b**) CD80 and (**c**) CD40 was increased after CpG stimulation but not significantly different between obese and lean mice. BMDCs were generated from the bone marrow of either obese or lean mice with GM-CSF (20 ng/mL) for six days. BMDCs were either left untreated or stimulated with CpG (0.25 µM) for 24 h and expression of activation markers analysed by flow cytometry. MFI of live CD11c^+^ cells is shown. The results are the mean + STD of 3 independent experiments performed in triplicate, with a total number of 3 mice/model. Statistical significance was determined using a One-way ANOVA test followed by Tueky’s multiple pairwise comparison. * *p* < 0.05, ** *p* < 0.01, **** *p* < 0.0001.

CpG stimulation led to significantly increased CD86 and MCHII expression levels in both lean and obese mice (Figure 2a,d). BMDCs from lean mice expressed significantly higher levels of CD86 and MHCII both in the un-stimulated state as well as following CpG stimulation, compared to BMDCs from obese mice.

CD80 expression in BMDCs from lean and obese mice was upregulated in response to CpG stimulation compared to the unstimulated DCs. However, there were no significant differences in CD80 expression observed between the BMDCs from obese and lean mice (Figure 2b).

Expression of CD40 in BMDCs from obese mice was significantly upregulated following CpG stimulation compared to the un-stimulated BMDCs. However, there was no up-regulation of CD40 in BMDCs from lean mice following CpG stimulation. There was also no difference in CD40 expression between the BMDCs from obese and lean mice in either the un-stimulated or the CpG stimulated groups (Figure 2c).

### 2.3. Breast Cancer and Colorectal Cancer Tumour Growth Rate in Lean and Obese Mice

**Figure 3:**
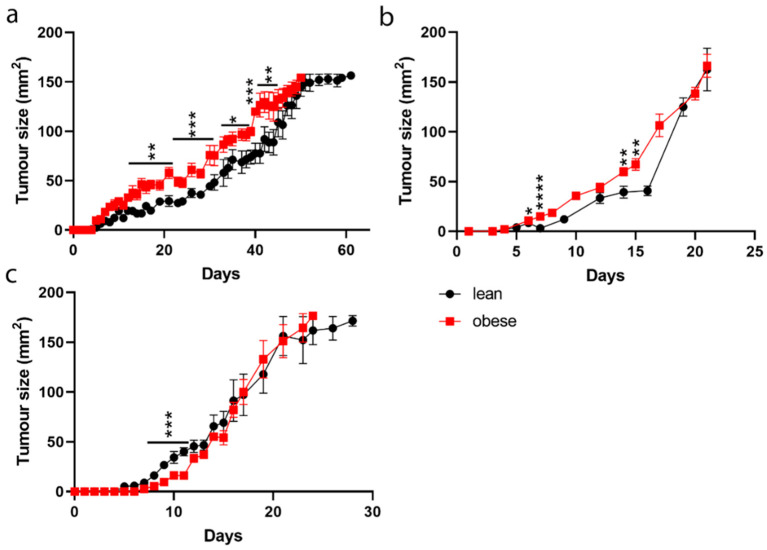
Tumour growth rate in lean and obese mice. (**a**) C57mg BC cells and (**b**) EO771.LMB metastatic BC cells were injected percutaneously into the mammary fat pads of lean and obese mice and tumour growth rate was recorded; (**c**) MC38 CRC cells were injected subcutaneously into the right flank of lean and obese mice and tumour growth rate was recorded. Results are the mean ± STD of *n* = 5 mice/group. Statistical significance was determined using a Wilcoxon rank-sum test. * *p* < 0.05, ** *p* < 0.01, *** *p* < 0.001, **** *p* < 0.0001.

Three different tumour cell lines were implanted into either lean or obese mice and their growth kinetics were observed. Non-metastatic BC from C57mg cells and metastatic BC from EO771.LMB cells were implanted percutaneously into the 3rd mammary fat while MC38 CRC cells were injected subcutaneously into the right flank. While there was no overall significant difference in tumour growth rate for any of the models, both the non-metastatic and metastatic BC showed prolong difference in the tumour size up until the tumours reached approximately 140 mm^2^. In both BC models the tumours grew faster in obese mice compared to lean mice (Figure 3a,b). In the CRC model, tumours grew faster in lean mice up to 50 mm^2^, with no difference in tumour size after that (Figure 3c).

### 2.4. Tumour-Infiltrating Lymphocytes Phenotyping

**Figure 4:**
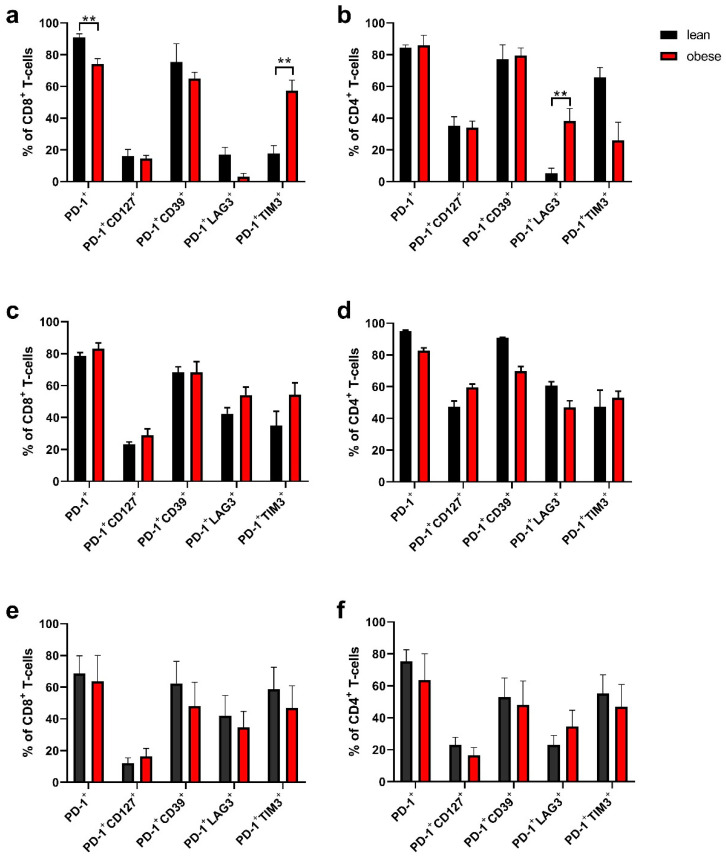
T-cell exhaustion of TILs in BC and CRC tumours in obese and lean mice. Tumours at 150 mm^2^ from (**a**,**b**) C57mg cells; (**c**,**d**) EO771.LMB cells and (**e**,**f**) MC38 cells were extracted from mice, cells were isolated using physical dissociation and purified using a Ficoll-Paque gradient. Cells were then stained for flow cytometry analysis. Results show the mean + STD of 5 mice/group. Statistical differences were determined by using an unpaired student’s *t*-test. ** *p* < 0.01.

Once tumours from the growth kinetic study reached a size of 150 mm^2^, individual mice were euthanised and the tumours were extracted to phenotype the TILs. Five tumours per group were collected in total and lymphocytes were analysed by flow cytometry. Expression of CD3, CD4 and CD8 were used to detect T cells and surface markers PD-1, LAG-3, Tim-3, CD127, and CD39 were used to phenotype T cells for exhaustion. Overall PD-1 expression in tumour-bearing lean and obese mice was high, indicating that there was some level of exhaustion in both CD8^+^ and CD4^+^ T cells. In the non-metastatic BC model (C57mg cells), lean mice had a higher percentage of CD8^+^PD-1^+^ cells compared to obese mice, while there was no difference in CD4^+^PD1^+^ cells (Figure 4a,b). While in the metastatic BC (EO771.LMB cells, Figure 4c,d) and CRC (MC38 cells, Figure 4e,f) models also high frequencies of PD1^+^ cells were observed, there were no differences between tumours from lean or obese mice (Figure 4).

Tumour-specific but terminally exhausted T cells can be characterised by being PD-1^+^ CD39^+^ [31]. There was no significant difference in expression of PD1^+^CD39^+^ cells in either CD4^+^ or CD8^+^ cells from obese and lean mice in any of the tumour models.

CD127, which is the α-chain of the IL-7 receptor and is important in long term persistence of T cells in the absence of an antigen, has reduced expression in exhausted T cells [29]. No difference in expression of PD1^+^CD127^+^ on CD4^+^ or CD8^+^ cells between cells from obese and lean mice was observed.

T cells which are terminally exhausted, express PD-1 as well as Tim-3 or LAG-3. A significantly higher frequency of CD8^+^PD1^+^TIM3^+^, as well as CD4^+^PD1^+^ LAG3^+^ were found in cells from obese versus lean mice in the non-metastatic BC model (Figure 4a,b). No other differences in cells from obese and lean mice were observed in any of the tumour models.

### 2.5. Frequency of Tumour-Infiltrating Lymphocytes

Apart from analysing the phenotype of exhausted TILs in the tumour models in lean and obese mice, we also investigated the infiltration frequency and spatial distribution of the TILs. Once tumours reached 150 mm^2^, they were extracted from the mice, formalin-fixed paraffin-embedded (FFPE), sectioned and stained for CD3 by immunohistochemistry. Slides were analysed for T-cell density in the whole tumour, intra- and peritumoural areas.

**Figure 5:**
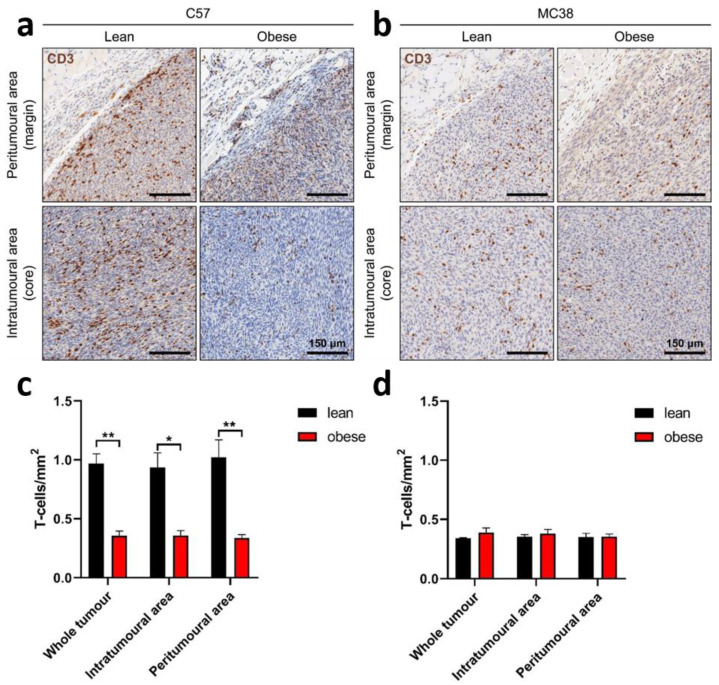
TIL frequencies in BC and CRC tumours. Tumours from (**a**,**c**) C57mg and (**b**,**d**) MC38 cells were extracted at 150 mm^2^, preserved by FFPE, sectioned for immunohistochemistry and stained with CD3. Images (**a**,**b**) show immunohistochemical staining for CD3 (DAB, brown) with a haematoxylin stain (blue) for the peritumoural (top row) and intratumoural (bottom row) CD3^+^ regions of C57mg and MC38 tumours. Results show the mean + STD of 5 mice/group. Statistical differences were determined by using an unpaired student’s *t*-test. * *p* < 0.05, ** *p* < 0.01.

In the C57mg BC model, a significantly enhanced infiltration of CD3^+^ T cells was observed in lean mice compared to obese mice (Figure 5a). These findings were observed in the whole tumour area as well as in the intra- and peritumoural area. No difference in T-cell frequency was seen between the different tumour areas.

In the MC38 CRC tumour model there was no difference in T-cell infiltrate between lean and obese mice either in the whole tumour area or the intra- or peritumoral area (Figure 5b).

### 2.6. Peripheral Blood Populations

To follow the expansion of immune cell populations in the blood during tumour growth, lean and obese mice grafted with MC-38 cells (CRC model) were tail bled every 7 days. Obese mice were only tail bled up to day 21, due to their enhanced tumour growth, while lean mice were tail bled until day 35 before reaching the humane endpoint. Samples were FACS stained and analysed via flow cytometry. Cell populations stained for were T cells (CD4^+^ and CD8^+^), B-cells (CD3^−^ B220^+^), inflammatory monocytes (CD3^−^ B220^−^ CD11b^+^ Ly6C^+^ Ly6G^−^), neutrophils (CD3^−^ B220^−^ CD11b^+^ Ly6C^+^ Ly6G^+^), and myeloid- and lymphoid-derived DCs (CD3^−^ B220^−^ CD11c^+^ CD11b^+^ or CD11b^−^ respectively). Figure S4 shows the gating strategy used to identify these cell types.

**Figure 6:**
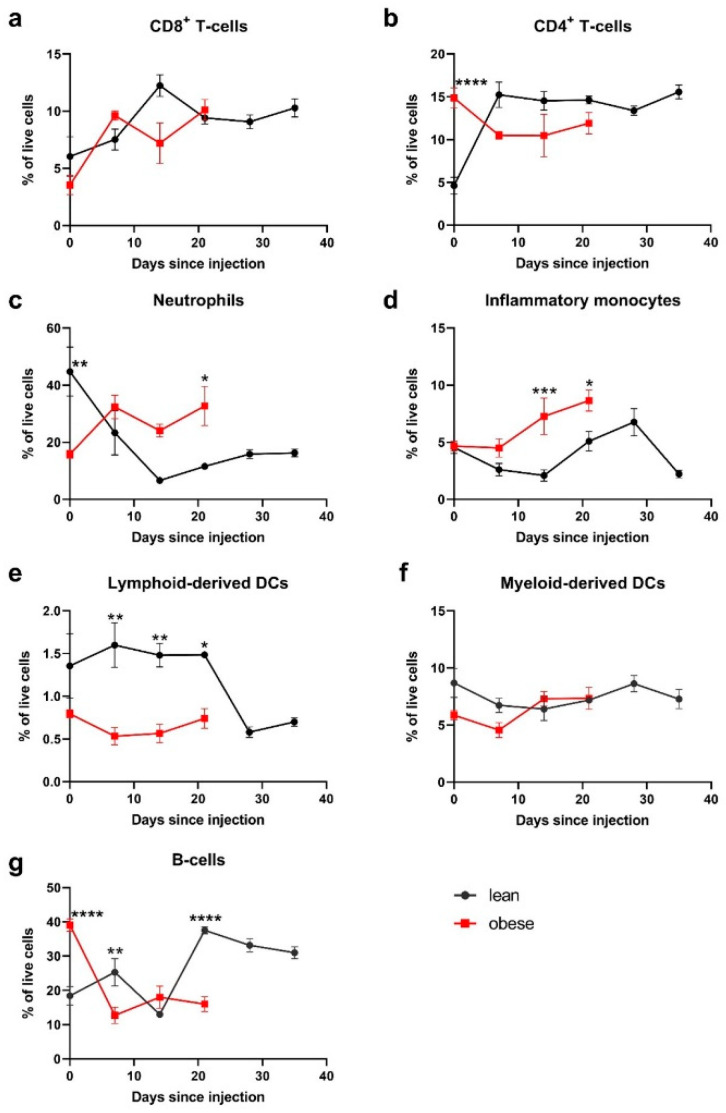
Analysis of immune cell populations in the blood of tumour-bearing mice. Lean and obese mice were tail-tipped every 7 days following MC38 cell engraftment and cells were analysed by flow cytometry for (**a**) CD8^+^ T cells; (**b**) CD4^+^ T cells; (**c**) neutrophils; (**d**) inflammatory monocytes; (**e**) lymphoid-derived DCs; (**f**) myeloid-derived DCs and (**g**) B-cells. Data are the mean ± STD from *n* = 5 mice/group. Statistical significance was determined using a two-way ANOVA followed by Sidak’s multiple comparison. * *p* < 0.05, ** *p* < 0.01, *** *p* < 0.001, **** *p* < 0.0001.

Differences in several cell populations were detected in lean and obese mice at differing time points. While the frequency of CD8^+^ T cells increased in both mouse types from baseline up to fourteen days post tumour cell grafting and remained steady at ~10% of live cells after this, no statistically significant differences were seen between the mouse types (Figure 6a).

CD4^+^ T cells were significantly higher in obese mice (*p* < 0.0001) before tumour engraftment; however, from day seven CD4^+^ frequencies dropped slightly in obese mice while in lean mice the population increased, with not more significant differences (Figure 6b). While lean mice initially had significantly higher frequencies of neutrophils at day 0 (*p* < 0.01), this decreased by day 7 so that obese mice had a trend of increased frequency, albeit no statistical difference was seen (Figure 6c).

Lean and obese mice had similar frequencies of inflammatory monocytes before tumour engraftment, from day seven obese mice had higher levels of inflammatory monocytes than lean mice, which was significant at day 14 (Figure 6d). Lean mice had significantly higher frequencies of lymphoid-derived DCs initially; however, this population decreased on day 28 (Figure 6e). Myeloid-derived DCs had similar frequencies in both lean and obese mice and did not fluctuate significantly throughout the experiment (Figure 6f).

Frequencies of B-cells were significantly higher in obese mice at day 0 (*p* < 0.0001); however, 7 and 21 days after tumour grafting, this was reversed to lean mice having a significantly higher frequency, while the frequency of B-cells in obese mice dropped following tumour engraftment (Figure 6g).

## 3. Discussion

The relationship between obesity and an increased risk of developing certain cancers has not been fully understood to date. Changes in the immune system, including an enhanced pro-inflammatory state and reduced T-cell antigenicity associated with obesity have been proposed as a possible link. Here we report on how cells of the innate and adaptive immune response differ both systemically and in the TME between obese and lean mice following BC and CRC tumour engraftment. In particular, we observed lower numbers of TILs and a higher frequency of exhausted T cells in the obese mice that is likely contributing to increased susceptibility for BC tumour growth.

Obesity causes increased lipid deposits in the thymus and bone marrow, disrupting their integrity and thereby altering the environment in which leukocytes develop [32]. In the bone marrow, this suppresses hematopoiesis and skews populations into producing more myeloid progenitor cells as opposed to lymphoid progenitor cells [33,34]. As observed in animal models and patients, a higher frequency of CD11b^+^ myeloid cells were confirmed in obese mice compared to the lean mice [35,36]. Additionally, MDSC expansion is associated with chronic inflammation, resulting in a decrease in differentiated DCs and macrophages, suppressing T-cell responses and inducing CTL tolerance to cancer cells [37]. We observed higher frequencies of both gMDSC and mMDSC in obese mice, indicating a more immunosuppressive environment compared to the lean mice, in line with literature from patients [38].

To investigate if the observed changes in immature myeloid cell populations would impact the generation of fully functional DCs, the phenotype of BMDCs was investigated, as they are crucial in forming a sustained and robust anti-tumour immune response. POUND mice were used as a model for obesity, they have a deletion mutation in exon two of the leptin receptor gene on chromosome four, resulting in it lacking the leptin receptor [39]. Leptin causes satiety, so this mutation renders mice unable to control the urge to eat. They quickly display features of metabolic syndrome from 8 weeks of age, mimicking the metabolic syndrome and obesity seen in people. Importantly, these mice, unlike the db/db mice or even diet-induced obese models remain pre-diabetic, allowing us to assess the impact of obesity without the added complication of diabetes.

Phenotypic analysis revealed that MHC II expression was decreased in BMDCs from obese mice compared to BMDCs from lean mice, both in the unstimulated format as well as following stimulation with the immune adjuvant CpG (TLR9 agonist). MHC upregulation is an important response following antigen detection, and it is vital to ensure increased antigen presentation following immune activation. Previous studies have found that MHC expression in DCs either isolated from splenocytes or generated as BMDCs from obese mice did not differ from that of wild type mice. However, these DCs were analysed following LPS stimulation, which is a TLR4 agonist, suggesting that the route of stimulation has an impact on cell activation in cells isolated from lean and obese mice [40,41]. Decreased MHC II expression as shown here may lead to impaired antigen presentation during the anti-cancer response as well as a decreased induction of T cell activation.

Previous studies in lean CD80 and CD86 knockout mice proposed that during T cell activation CD86 is the initial ligand for CD28, due to its rapid and high expression on APCs, while CD80 appears to be the more potent ligand for CD28 subsequently [42]. Thus, decreased CD86 but not CD80 may imply that DCs from obese mice may have impaired initial T cell co-stimulation, while subsequent potent co-stimulation may not be impacted.

The activation marker CD40 functions by binding to CD40L on licensed T cells, resulting in cytokine production by DCs, induction of co-stimulatory molecule cell surface expression and promoting antigen cross-presentation [43]. This DC maturation is required for effective T cell differentiation and activation [43]. While CpG activated BMDCs from obese mice expressed lower CD40, this was not statistically different. However, decreased expression of CD40 may result in impaired DC maturation leading to a downstream impact of T cell responses.

These initial experiments of phenotyping BM and activated BMDC cells confirmed that cell frequencies and changes in the ability to mount a strong immune response are impacted in obese mice as compared to lean mice.

While focusing on these specific cell types in vitro is an important indicator of anti-cancer immune responses, the following experiments focused on establishing any differences in in vivo tumour models, factoring in other host variations such as the full range of immune cells, cytokines, and the tumour microenvironment. As anticipated from the in vitro data, BC tumours in obese mice grew faster than in lean mice, confirming observations in various patient cohorts [44,45]. The lack of difference in overall survival time between obese and lean mice may be attributed to the immune system in lean mice keeping the tumour better in check while it was small; however, once the tumour reached a certain size in both models the tumour could escape. The minimal difference in the tumour growth rate in the CRC model, with larger tumours in the lean mice up to 50 mm^2^, may be due to the difference in the tissue environment. While the BC tumours growing orthotopically in the mammary fat pad may have been more affected by the adipose tissue directly surrounding them, this effect could have been mitigated by the subcutaneous position of the CRC tumours [46].

To understand changes to the tumour growth rate further, TILs from extracted tumours were analysed both for their total number and their potential exhausted phenotype. When T cells are continuously stimulated by antigens, such as in the TME of our BC and CRC tumour models, they convert to an exhausted form. Once exhausted, the effective anti-tumour immune response becomes reduced and the numbers of antigen-specific T cells are depleted [29]. Exhausted T cells have an upregulated expression of several inhibitory immune checkpoint receptors such as PD-1 once initially exhausted, and Tim-3 and LAG-3 when terminally exhausted [29]. The higher frequency of early stage exhausted CD8^+^ T cells (PD-1^+^ TIM3^+^) and later stage exhausted CD4^+^ T cells (PD-1^+^ LAG3^+^) in obese mice may offer an explanation for the faster tumour growth rate in the non-metastatic BC model. Viewing this in combination with the increased total CD3^+^ T-cell number in lean mice compared to obese mice analysed by immunohistochemistry suggests that both the number of T cells as well as the exhausted phenotype of T cells contribute to difference in tumour growth between obese and lean mice, at least in our non-metastatic BC model.

The lack of consistent differences between the phenotype of TILs from obese and lean mice in the metastatic BC and CRC tumour models for tumour-specific and terminally exhausted T cells may be due to the study only looking at tumours that reached the 150 mm^2^ endpoint and hence already achieved immune escape. Preliminary studies of analysing TIL phenotype on day 7, 14 and 21 after engraftment of non-metastatic BC tumours in mice showed that there is an increase in frequency of tumour-specific T cells as the tumour burden increases; however, these cells also become more exhausted (Figure S3). Similar to the results for the TIL phenotyping, there were no differences in the number of CD3 cells in the CRC mouse models. The use of a subcutaneous model, rather than an orthotopic one for CRC, may have contributed to the lack of difference in tumour growth kinetics, TIL phenotype and TIL numbers, as it lacks the specific immune microenvironment of the gastrointestinal tract [47].

As no differences in the tumour growth rate and TILs were observed for the CRC model, analysis of immune cell populations in the blood were used to investigate whether there were any other, peripheral changes. Consistent with patient data where obesity has been linked to a higher total lymphocyte count, the obese mice had higher levels of CD4^+^ T cells before tumour engraftment compared to lean mice, while the level of CD8^+^ T cells was similar in both mouse types [48,49].

The lower levels of neutrophils and lymphoid-derived DCs in obese mice within the first weeks of tumour growth may reduce the ability of the immune system to mount a strong anti-cancer immune response, as these cells are crucial for the presentation of antigens to cells of the adaptive immune system. Furthermore, the levels of inflammatory monocytes increased more rapidly in obese mice, which may be associated with suppression of anti-tumour immunity. This may indicate that while there was no discernible difference in the tumour growth rate in the CRC model, the underlying inflammation brought about by obesity meant that these mice were unable to create as strong an anti-tumour immune response against the tumour cells as their lean counterparts.

## 4. Materials and Methods

### 4.1. Mice

Specific-pathogen-free C57BL/6NCrl (lean) mice were sourced from the Hercus Taieri Research Unit, University of Otago, Dunedin, New Zealand. POUND (obese: C57BL/6NCrl-Lepr^db-lb^/Crl) mice on a C57BL/6 background were gifted from Dr Jacquie Harper (Malaghan Institute of Medical Research, Wellington, New Zealand), but can be obtained from Charles River Laboratories (USA) and maintained in-house. Female and male mice, between 2 and 7-months of age, were used for experiments. Female and male C57BL/6 weighed 23.53 ± 1.85 g and 32.14 ± 5.17 g, respectively. Female and male obese POUND mice weighed 59.07 ± 12.94 g and 57.94 ± 10.21 g. Each experimental protocol was approved prior to commencement by the University of Otago Animal Ethics Committee (AEC18/31 and AUP18-216). Mice were housed under a 12-h light/dark cycle with unlimitedaccess to food and water and were randomly assigned to a treatment group prior to tumour cell administration. All animals were euthanized by cervical dislocation.

### 4.2. Phenotyping of Bone Marrow Cells

Bone marrow cells were harvested from the hind legs of female C57Bl/6 and POUND mice and processed to single cell suspensions. An aliquot was stained with the LIVE/DEAD yellow fixable stain kit to exclude dead cells and with fluorophore-conjugated antibodies against B220 (PE/CF594), CD11b (APC), CD11c (PE), CD3 (AlexaFluor700), Ly6C (PE/Cy7) and Ly6G (FITC). Stained cells were run on a Beckman Coulter Gallios and analysed using the Kaluza analysis software version 1.2. A complete list of antibodies used for flow cytometry can be found in Table S1.

### 4.3. Generation and Activation of BMDCs

BMDCs were prepared from bone marrow cells harvested from female C57Bl/6 and POUND mice as previously described [50]. Briefly, femurs and tibiae from euthanized mice were isolated and the red blood cells were lysed with ammonium chloride. Single bone marrow cells were seeded at 0.5 × 10^6^ cells/mL in complete Iscove’s Modified Dulbecco’s Medium (cIMDM, + 5% foetal calf serum, 1% Penicillin/Streptomycin, 0.1% 2-mercaptoethanol) supplemented with 20 ng/mL GM-CSF and incubated at 37 °C and 5% CO_2_. BMDCs were harvested on day 6 of culture and seeded into a 24-well plate at 1 × 10^6^ cells/mL. BMDCs were either left untreated or stimulated with CpG (0.25 µM) for 24 h. Cells were harvested and the expression of cell surface markers CD40 (PE), CD80 (Pacific Blue), CD86 (PE/Cy7) and MHCII (APC/Cy7) on live, CD11c^+^ BMDCs was assessed by flow cytometry.

### 4.4. Tumour Cell Lines and Tumour Challenges

C57mg cells were kindly provided by Prof. Sandra Gendler, Mayo Clinic, Arizona, USA and EO771.LMB were kindly provided by Prof. Robin Anderson, Olivia Newton-John Cancer Research Institute, Australia [51]. C57mg and EO771.LMB cells were injected percutaneously into the second mammary fat pad and MC38 cells subcutaneously into the left flank of lean and obese mice at 1 × 10^5^ cells/mouse, *n* = 10 mice per cell line. Tumour size was measured every two days using calipers, and when the tumour reached 150 mm^2^, mice were culled by cervical dislocation.

### 4.5. Assessment of Tumour-Infiltrating Lymphocytes

Tumours at 150 mm^2^ from lean and obese mice were extracted for tumour infiltrating lymphocyte (TIL) analysis. Five tumours per group were fixed in formalin for CD3 immunohistochemistry (Histology Unit, University of Otago). Images were taken with Aperio CS2 and analysed using Fiji-ImageJ. To measure the area of each cross-section, a freehand selection line was drawn around the border of each tumour. Tumours were then split into inner and outer tumour regions, defined by a 250 µm width margin from the outer border. Area measurements were taken at this point. The different components of the histological stain were then separated, and the DAB component selected. The image was converted to black-and-white and individual cells defined in order to be able to be read and counted by the software. This process was repeated for whole tumour, inner tumour and outer tumour images.

The other five tumours were processed into single cell suspensions by physical maceration and filtration through 70 μm cell strainers, followed by a Ficoll (Ficoll-Paque PLUS density gradient media, GE Healthcare) gradient. TILs were stained with Zombie-Yellow Live/Dead Stain, treated with CD16/CD32 Fc blocking antibody, and then stained with PD-1 (FITC), LAG3 (PE), CD127 (PE/CF594), CD39 (PE/Cy7), TIM3 (APC), CD8 (AF700), CD4 (APC/H7) and CD3 (BV421). Fluorescence was measured using a Gallios flow cytometer and analysed using Kaluza software (Beckman Coulter, Brea, CA, USA).

### 4.6. Phenotyping of Peripheral Blood Immune Cell Populations

Blood from lean and obese mice injected with MC-38 cells was collected. Tails of five mice were tipped and 4–5 drops of blood collected into 1 mL Alsever’s solution before injection of tumour cells. The blood of five mice per group was then collected weekly until the end of the trial. Samples were centrifuged for 5 min at 350× *g* at 4 °C and red blood cells were lysed with ammonium chloride. Cells were stained with Zombie-Yellow Live/Dead Stain, treated with CD16/CD32 Fc blocking antibody, and then stained with Antibodies against Ly6G (FITC), Ly6C (PE/Cy7), CD11c (PE), B220 (PE-Dazzle), CD8 (AF700), CD4 (APC/H7) and CD3 (BV421) (see Table S1).

### 4.7. Statistical Analysis

Data are shown as mean ± STD. The statistical significance between values was assessed using one-way ANOVA followed by post-hoc Tukey’s pairwise comparison (BMDC activation), unpaired student’s *t*-test (BM cell characterisation, TIL phenotyping and frequency). Longitudinal peripheral immune cell populations were compared using two-way ANOVA followed by Sidak’s multiple comparison. Tumour growth rates were compared by using the Wilcoxon rank-sum test. Statistical analysis was performed using GraphPad Prism version 8.00.

## 5. Conclusions

In summary our findings describe a range of systemic effects of obesity on the immune system in both naïve and tumour-bearing mice. Immune cells from obese mice were shown to have elevated levels of myeloid cells and generate lower levels of activation markers following adjuvant stimulation compared to cells from their lean counterparts. In the tumour setting, lower total numbers of TILs and a higher frequency of exhausted T cells in obese mice support the enhanced tumour growth rate of BC tumours in obese mice. These findings provide an explanation for the higher tumour burden observed in obese patients with BC and highlight that systemic changes to the immune system are clinically relevant for these patients.

## Data Availability

Data supporting the results may be retrieved from the authors upon request.

## Supplementary Materials

The following are available online at https://www.mdpi.com/article/10.3390/ijms22168803/s1, Figure S1: Gating strategy for bone marrow cell population, Figure S2: Gating strategy for BMDC, Figure S3: TIL phenotype metastatic BC time course, Figure S4: Gating strategy for immune populations in the blood, Table S1: Flow cytometry antibody list.
