# Supplementary material for: Obesity Has a Systemic Effect on Immune Cells in Naïve and Cancer-Bearing Mice
Source: Int J Mol Sci. 2021 Aug 16;22(16):8803. doi: 10.3390/ijms22168803 (PMC8395769; doi:10.3390/ijms22168803)
Supplement: Supplementary file 1 [file ijms-22-08803-s001.zip › Table S1 Antibodies.pdf]

**Anti-mouse antibodies**	**Fluorophore**	**Concentration**	**Dilution**	**Clone**	**Supplier**
**CD11b**	APC	0.2mg/mL	1:400	M1/70	Biolegend
**CD11c**	PE	0.2mg/mL	1:100	N418	Biolegend
**CD127**	PE/CF594	0.2mg/mL	1:100	5B/199	BD Biosciences
**CD16/32 (FcR Block™)**	None	0.5mg/mL	1:200	2.4G2	BD Biosciences
**CD223 (LAG3)**	PE	0.2mg/mL	1:100	C9B7W	BD Biosciences
**CD279 (PD-1)**	FITC	0.5mg/mL	1:100	29F.1A12	Biolegend
**CD3**	BV421	0.2mg/mL	1:200	17A2	Biolegend
**CD3**	AF700	0.5mg/mL	1:200	500A2	BD Biosciences
**CD366 (TIM3)**	APC	0.2mg/mL	1:100	RMT3-23	Biolegend
**CD39**	PE/Cy7	0.2mg/mL	1:100	Duha59	Biolegend
**CD4**	APC/H7	0.2mg/mL	1:200	GK1/5	BD Biosciences
**CD40**	PE	0.2mg/mL	1:200	3/23	Biolegend
**CD80**	Pacific Blue	0.5mg/mL	1:200	16-10AI	Biolegend
**CD86**	PE/Cy7	0.2mg/mL	1:800	GL-1	Biolegend
**MHCII**	APC/Cy7	0.2mg/mL	1:800	M5/II4.15.2	Biolegend

**CD45R/B220**	PE-CF594	0.2mg/mL	1:100	RA3-6B2	BD Biosciences
**CD8α**	AF700	0.5mg/mL	1:200	53-6.7	Biolegend
**Live/Dead**	Zombie Yellow™		1:200		Biolegend
**Ly-6C**	PE/Cy7	0.2mg/mL	1:400	HK1.4	Biolegend
**Ly-6G**	FITC	0.5mg/mL	1:200	1A8	BD Biosciences
