# Supplementary figures and images for: Obesity Has a Systemic Effect on Immune Cells in Naïve and Cancer-Bearing Mice
Source: Int J Mol Sci. 2021 Aug 16;22(16):8803. doi: 10.3390/ijms22168803 (PMC8395769; doi:10.3390/ijms22168803)

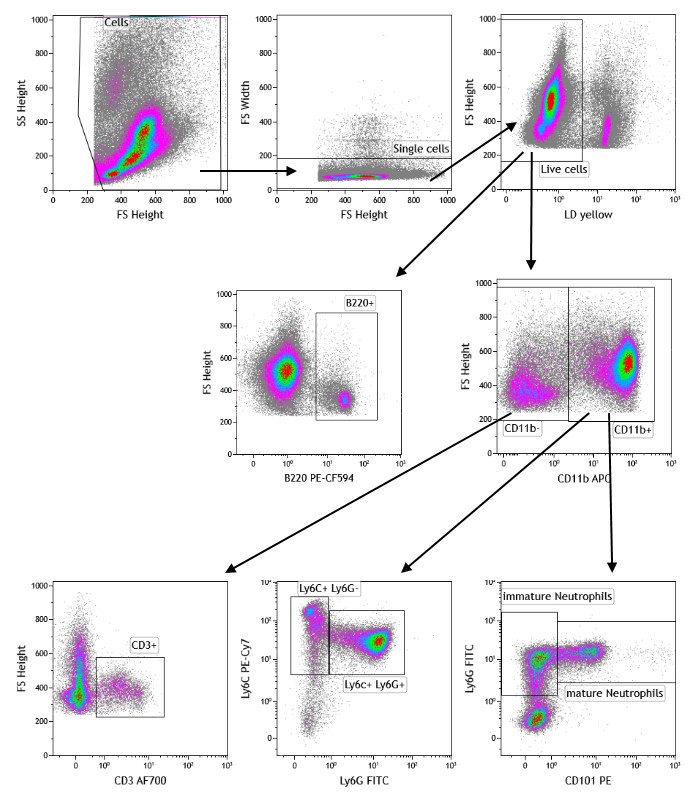

Supplement: Supplementary file 1 [file ijms-22-08803-s001.zip › Fig S1 Gating strategy BM cells.jpg]

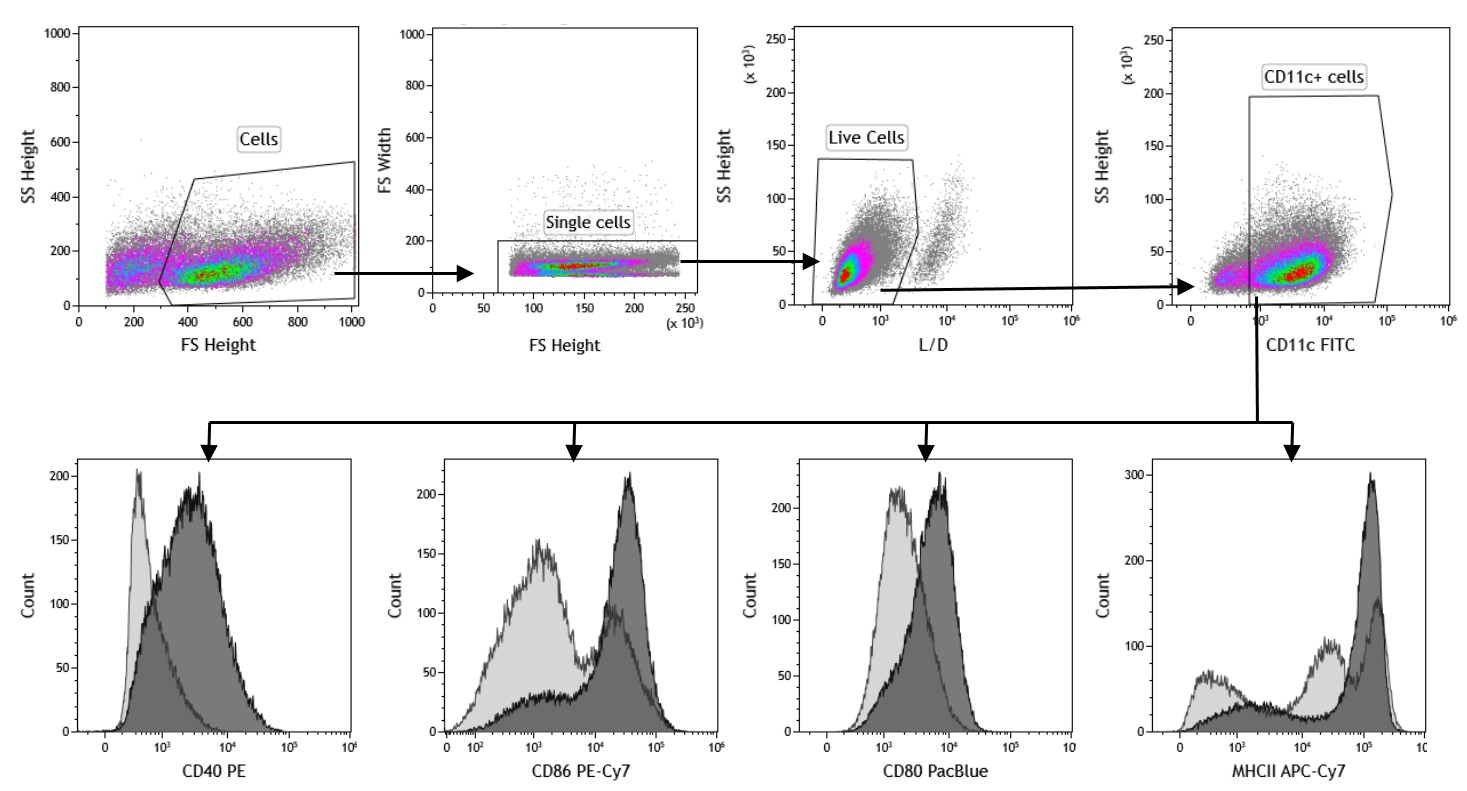

Supplement: Supplementary file 1 [file ijms-22-08803-s001.zip › Fig S2 Gating strategy BMDCs.jpg]

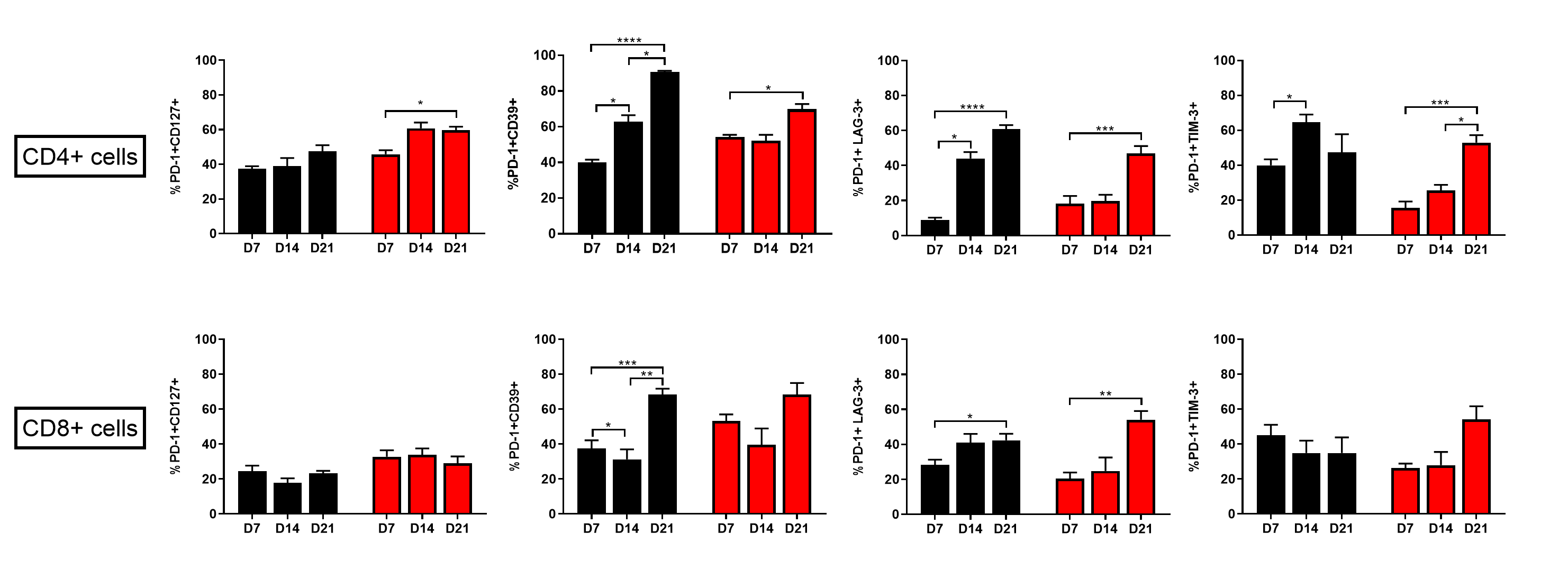

Supplement: Supplementary file 1 [file ijms-22-08803-s001.zip › Fig S3 TILs mBC.tif]

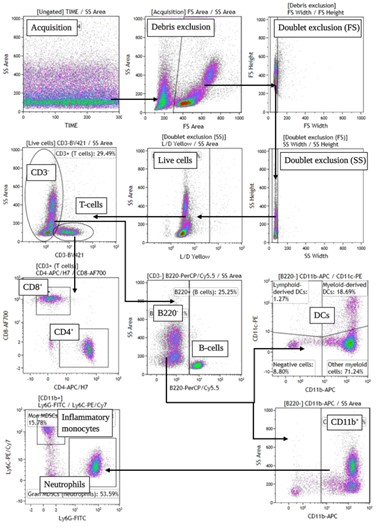

Supplement: Supplementary file 1 [file ijms-22-08803-s001.zip › Fig S4 Gating strategy blood cells.jpg]
